# Supplementary material for: MetaQTL: a package of new computational methods for the meta-analysis of QTL mapping experiments
Source: BMC Bioinformatics. 2007 Feb 8;8:49. doi: 10.1186/1471-2105-8-49 (PMC1808479; doi:10.1186/1471-2105-8-49)
Supplement: Additional File 7 — MetaQTL Package : jar file and tutorial. This Zip archive contains both the MetaQTL JAR file and the files of the tutorial. [file 1471-2105-8-49-S7.zip › org.inra.metaqtl/doc/org/metaqtl/graph/QtlUnit.html]

QtlUnit


|  |  |  |
| --- | --- | --- |
| **Overview** \| **Package** \| **Class** \| **Use** \| **Tree** \| **Deprecated** \| **Index** \| **Help** | |  |
| **PREV CLASS**   NEXT CLASS | **FRAMES**    **NO FRAMES**     **All Classes** |  |
| SUMMARY: NESTED \| FIELD \| CONSTR \| METHOD | DETAIL: FIELD \| CONSTR \| METHOD |  |


---


## org.metaqtl.graph Class QtlUnit

```
java.lang.Object
  org.metaqtl.graph.QtlUnit
```

---

``` public class QtlUnit extends java.lang.Object ```

---

| **Field Summary** | |
| --- | --- |
| `(package private)  double` | `from` |
| `(package private)  java.lang.String` | `name` |
| `(package private)  double[]` | `partition` |
| `(package private)  double` | `pos` |
| `(package private)  double` | `to` |
| `(package private)  double` | `width` |
| `(package private)  double` | `x` |


**Constructor Summary**

`QtlUnit()`


**Method Summary**

**Methods inherited from class java.lang.Object**

`clone, equals, finalize, getClass, hashCode, notify, notifyAll, toString, wait, wait, wait`

**Field Detail**

### x

```
double x
```

---


### pos

```
double pos
```

---


### from

```
double from
```

---


### to

```
double to
```

---


### width

```
double width
```

---


### name

```
java.lang.String name
```

---


### partition

```
double[] partition
```


**Constructor Detail**

### QtlUnit

```
public QtlUnit()
```


---


|  |  |  |
| --- | --- | --- |
| **Overview** \| **Package** \| **Class** \| **Use** \| **Tree** \| **Deprecated** \| **Index** \| **Help** | |  |
| **PREV CLASS**   NEXT CLASS | **FRAMES**    **NO FRAMES**     **All Classes** |  |
| SUMMARY: NESTED \| FIELD \| CONSTR \| METHOD | DETAIL: FIELD \| CONSTR \| METHOD |  |


---
